# Development and Evaluation of Ferrite Core Inductively Coupled Plasma Radio Frequency Ion Source for High-Current Ion Implanters in Semiconductor Applications

**DOI:** 10.3390/s24155071

**Published:** 2024-08-05

**Authors:** Jong-Jin Hwang, Hyo-Jun Sim, Seung-Jae Moon

**Affiliations:** Department of Mechanical Convergence Engineering, Hanyang University, Seoul 04763, Republic of Koreagywns7078@hanyang.ac.kr (H.-J.S.)

**Keywords:** implanter ion source, ferrite core inductively coupled plasma radio frequency ion source, ion source lifetime improvement, RF ion source

## Abstract

This study presents the development of a ferrite core inductively coupled plasma (ICP) radio frequency (RF) ion source designed to improve the lifetime of ion sources in commercial ion implanters. Unlike existing DC methods, this novel approach aims to enhance the performance and lifetime of the ion source. We constructed a high-vacuum evaluation chamber to thoroughly examine RF ion source characteristics using a Langmuir probe. Comparative experiments assessed the extraction current of two upgraded ferrite core RF ion sources in a commercial ion implanter setting. Additionally, we tested the plasma lifetime of the ICP source and took temperature measurements of various components to verify the operational stability and efficiency of the innovative design. This study confirmed that the ICP RF ion source operated effectively under a high vacuum of 10^−5^ torr and in a high-voltage environment of 30 kV. We observed that the extraction current increased linearly with RF power. We also confirmed that BF_3_ gas, which presents challenging conditions, was stably ionized in the ICP RF ion sources.

## 1. Introduction

Most ion implanters utilize indirectly heated cathode (IHC) direct current (DC) ion sources. Over the recent 20 years, considerable progress has been made in extending the lifetime and enhancing the stability of these systems [1,2,3,4,5]. Despite these advances, issues such as filament breakage due to cathode punch-through and tungsten deposition from the halogen cycle persist, necessitating further improvements [6,7,8,9]. This study addresses the problem of cathode hole formation due to thermal electron emission by developing an ion source based on the remote plasma source (RPS) concept, utilizing a ferrite core [10,11,12,13,14,15]. The RPS structure was anticipated to improve the ion source’s lifetime by preventing electrodes from being exposed to plasma, which typically causes erosion and shortens the operational ion source lifetime [16,17,18,19,20]. We selected the ferrite core inductively coupled plasma (ICP) source because we expected it to operate stably, as demonstrated by the RPSs commonly used in etching and thin film processes within semiconductor equipment. Additionally, we anticipated that the ferrite core ICP source would generate plasma with a simple and efficient structure as it transmits external radio frequency (RF) power through the ferrite core without needing electrodes directly inside the plasma. We excluded other ICP methods besides the ferrite core method at the design stage because they were unsuitable for manufacturing with the existing parts (extraction electrode, source bushing) of the currently used ion implanter. RPS systems utilize a high gas flow rate to sustain plasma ignition and operation. However, achieving stable plasma ignition under a low gas flow of less than 5 square cubic cm per min (sccm) and high-vacuum conditions typical of ion implantation processes posed a significant challenge. The low gas flow rates can lead to difficulties in maintaining the necessary plasma density for effective ion generation [21]. To overcome this, our ferrite core RPS design incorporated specific adjustments to facilitate plasma ignition and sustainment under these stringent conditions. After achieving stable plasma ignition, we enhanced the ICP efficiency by utilizing ferrite cores, which ensured stable plasma operation even at reduced gas flow rates. An extensive experimental design was considered to evaluate the performance of this new ion source configuration, focusing on parameters such as ion density, plasma stability, and operational lifetime under various flow rates and vacuum conditions. The performance of the ferrite core ICP source was tested using a test chamber. The extraction current was compared to confirm the ion density by mounting it on the ion source of a commercial ion implanter under optimal conditions [22,23,24,25,26,27,28,29,30]. The extraction current of the argon beam was evaluated, and the process gas, BF_3_, was additionally evaluated to compare the two gases. We selected BF_3_ for comparison with argon because process gases combined with hydrogen, such as AsH_3_ and PH_3_, are easy to ionize, whereas gases combined with fluorine, such as BF_3_ and GeF_4_, are much more difficult to ionize. Therefore, we chose BF_3_, the most challenging gas to ionize, to demonstrate the effectiveness of our ICP RF source.

## 2. Materials and Methods

### 2.1. Ferrite Core ICP RF Ion Source Head

**Figure 1:**
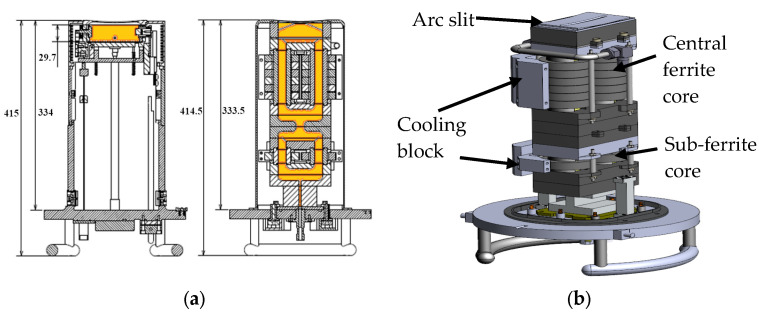
(**a**) Cross-sectional view left: IHC DC source; right: ferrite core ICP RF source (The orange color indicates the area where plasma occurs.); (**b**) ver. 1.1 ICP RF source side view (half side cooling block removed).

Figure 1a illustrates the design of the ion source, which matches the dimensions of the IHC DC source used in commercial ion implanters. The arc slit’s radius of curvature is identical to that of the extraction electrode, which is responsible for ion beam formation and fits precisely within the vacuum chamber where the ion source is mounted. The area expressed in orange color inside the cross-sectional view indicates where plasma is generated.

Figure 1b shows the side view of the ion source, with half of the cooling block removed to reveal the internal structure. The ferrite core is divided into two main components: the sub-ferrite core and the central ferrite core. The sub-ferrite core is designed to facilitate easy plasma ignition and improve the mobility of process gas under high-vacuum conditions by receiving 50% of the power delivered through a parallel connection of a 13.56 MHz RF generator (REXi-3K, RFPT, Suwon-si, Gyeonggi-do, Republic of Korea). This core ensures stable plasma generation even under challenging low-pressure conditions. The central ferrite core comprises six individual ferrite cores arranged in two parallel cylindrical structures.

This configuration is optimized to enhance the efficiency of the RF ion source, improving ion density and stability. The detailed design ensures compatibility with the existing infrastructure of high-current ion implanters, enabling seamless integration and operation.

### 2.2. Ferrite Core ICP Source Head Improvement

When applying an ICP source using a ferrite core to the implanter, the primary consideration was creating a structure capable of operating under a vacuum of less than 10^−5^ torr. The development process involved multiple iterations, aiming to improve the design for better performance and reliability.

In ver. 1.0, this initial version was designed to assist plasma ignition by placing a 100 V DC ignitor aid between the gas inlet and the ferrite core. The test setup involved using argon at a flow rate of 5 sccm under a vacuum level of 3.0 × 10^−5^ torr. The results showed that plasma ignition was achieved after more than 5 s of applying the 100 V DC. This demonstrated the feasibility of using a DC ignition aid to initiate plasma in high-vacuum conditions.

In ver. 1.1, the goal was to enhance the plasma ignition process and eliminate ignition components by connecting a sub-ferrite core in parallel with the existing central ferrite core for RF power delivery. This approach aimed to concentrate power delivery and facilitate plasma ignition without requiring external DC power assistance. Testing with argon at a reduced flow rate of 3 sccm confirmed that plasma ignition was achievable without the DC ignition aid, indicating a more efficient design. Ver. 1.1 also addressed heat management issues observed in earlier versions by introducing a cooling block. This block allowed process cooling water (PCW) to flow at 10 L per min (LPM), effectively dissipating the heat generated by the ferrite core. This improvement eliminated the need for the DC ignition aid while ensuring stable operation by controlling the temperature of the ferrite core.

In ver. 1.2, RF power was applied through seven pairs of central ferrite cores, with an additional pair of ferrite cores added to enhance ion density. The linear connection between the plasma-generated sub-loop and the central loop was streamlined to minimize plasma collisions with the chamber wall. Additionally, for improved cooling efficiency, we switched to a cooling block that covers all the central ferrite cores, including the sub-ferrite core. The ceramic tubes of the central loop were split into two parts to improve assembly convenience and reduce manufacturing costs.

### 2.3. Vacuum Chamber Design and Setup

A vacuum chamber was designed and constructed to measure the ignition and density of plasma in the ICP RF source head. The chamber was designed to replicate the dimensions and operational conditions of the source chamber used in commercial ion implanters. The vacuum chamber dimensions are 405.5 mm in width, 755.5 mm in length, and 335 mm in height. This configuration ensured that the experimental setup closely mimicked the ion source’s operational environment.

A turbo pump (MAG W 3200CT, LEYBOLD, Cologne, Germany) maintained high-quality vacuum conditions. The vacuum level was continuously monitored using a hot cathode ion gauge and a vacuum gauge controller (IVC2300, ISVT, Yongin-si, Republic of Korea). The system could maintain a vacuum level of 5.0 × 10^−6^ torr or lower, adjustable to 5.0 × 10^−5^ torr based on the gas flow rate. The flow rate of the process gas was controlled using a mass flow controller (MFC) (IMC1300, ISVT, Yongin-si, Gyeonggi-do, Republic of Korea). Precise gas flow control is crucial for maintaining stable plasma conditions.

A source magnet precisely installed to match the magnetic flux in the center of the plasma increased the plasma density of the ion source. The magnetic field strength was adjusted between 0 and 0.01 Tesla using a DC power supply (EX 600-2, ODA Technologies, Incheon Metropolitan City, Republic of Korea).

A double Langmuir probe (ALP-150, IMPEDANS, Dublin, Ireland) was installed by connecting the probe bellows to the back of the source chamber to compare the internal plasma characteristics of ion source heads.

## 3. Results and Discussions

### 3.1. Measurement of Ion Density Using Langmuir Probe and Extraction Current at Commercial Implanter

**Figure 2:**
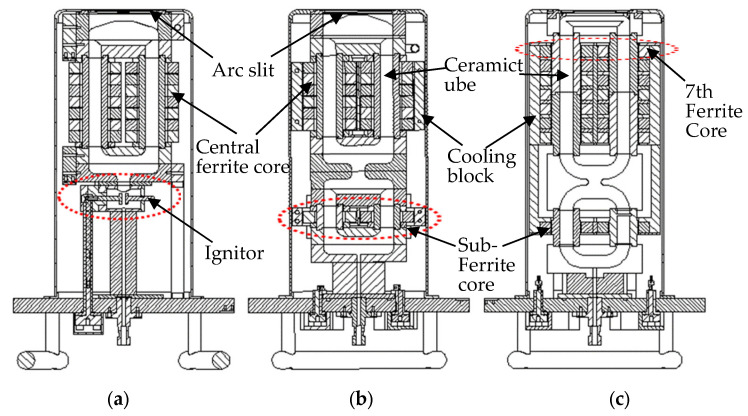
Ferrite core ICP source head cross-sectional view. (**a**) Ver. 1.0 (red dotted circle: ignitor), (**b**) ver. 1.1 (red dotted circle: added sub-ferrite core), and (**c**) ver. 1.2 (red dotted circle: added 7th ferrite core).

**Figure 3:**
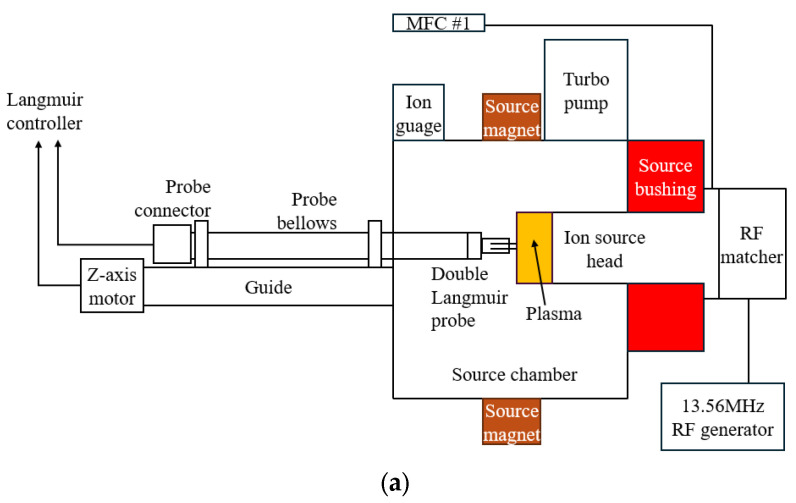

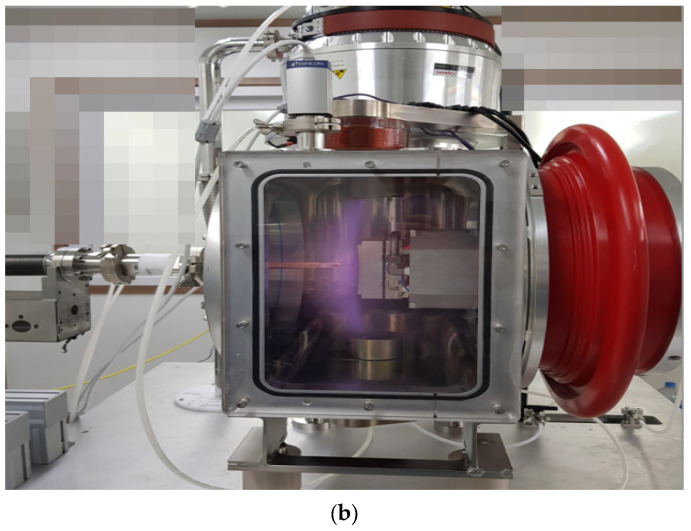
(**a**) System diagram of the test chamber and Langmuir probe (The orange color indicates the area where plasma occurs); (**b**) measurement of the ferrite core ICP source argon plasma ion density in the test chamber.

**Figure 4:**
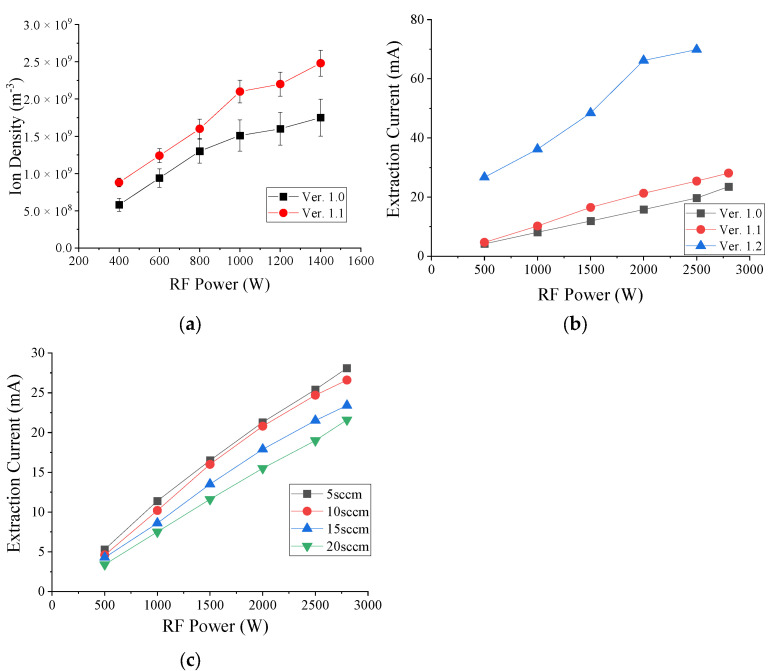
(**a**) Langmuir probe ion density measurement of ver. 1.0 and 1.1; (**b**) extraction current measurement ver. 1.0, 1.1, and 1.2; (**c**) comparison of extraction current compared to RF power according to gas flow rate change in ver. 1.1.

Ver. 1.0 and 1.1 (Figure 2a,b) were installed in the test chamber depicted in Figure 3b, and the plasma density of argon gas was measured at 5 sccm. Figure 4a presents the data obtained as the RF power increased incrementally from 400 to 1400 W. Notably, ver. 1.0 features seven pairs of central ferrite cores, whereas ver. 1.1 includes six pairs of central ferrite cores and one pair of sub-ferrite cores, marking a significant difference between the two versions. Ver. 1.0’s ion density at 400 W was 5.8 × 10^8^ m^−3^, while ver. 1.1’s increased by 52% to 8.8 × 10^8^ m^−3^. At 600 W, the ion density of ver. 1.0 increased by 32% to 1.24 × 10^9^ m^−3^ compared to 9.4 × 10^8^ m^−3^. This trend continued as the RF power increased, with a 23% increase at 800 W, 39% at 1000 W, 38% at 1200 W, and 42% at 1400 W. The consistent rise in ion density with increasing RF power highlights a clear correlation.

It was verified in the test chamber that plasma initiation and stabilization could be maintained for 2 h or longer. Subsequently, the extraction current was measured using a commercial ion implanter (OPTIMA HDx, AXCELIS, Beverly, MA, USA). In the ion source, plasma exists, and when applying 10 kV or higher energy to the ion source and extraction electrode, it transitions into an ion beam form [31,32,33,34]. The ion density can be determined by measuring the current at the extraction electrode.

In Figure 4b, vers. 1.0, 1.1, and 1.2 were mounted on a commercial implanter, and the extraction current was measured at 5 sccm of argon gas. RF power was gradually increased from 500 to 2800 W in 500 W increments using a 3000 W RF generator and adjusted to 2800 W due to power constraints. At 500 W, the extraction current of ver. 1.0 was 4.2 mA, ver. 1.1 increased by 12% to 4.7 mA, and ver. 1.2 increased by 635% to 26.7 mA. At 1000 W, ver. 1.1 increased by 26% to 10.2 mA compared to 8.1 mA in ver. 1.0, and ver. 1.2 increased by 446% to 36.2 mA. At 1500 W, ver. 1.1 increased by 39% and ver. 1.2 increased by 406%. At 2000 W, ver. 1.1 increased by 35% and ver. 1.2 increased by 418%. At 2500 W, ver. 1.1 increased by 29% and ver. 1.2 increased by 354%. At 2800 W, ver. 1.1 increased by 20%. After installing sub-ferrite cores in ver. 1.1, the extraction current continued to increase, improving by an average of 27% compared to ver. 1.0. Ver. 1.2 showed an average density increase of 452% after adding the 7th ferrite core and streamlining the area where plasma is generated.

In Figure 4c, the extraction current was measured by increasing the argon gas flow rate from 5 to 20 sccm in increments of 5 sccm while ver. 1.1 was installed. As a result, it was confirmed that the extraction current decreased as the gas flow rate increased. RF power was measured by increasing it in 500 W increments from 500 to 2800 W. The extraction current was 5.3 mA at 500 W and 5 sccm, and the flow rate decreased by 13%, 19%, and 36% as the flow rate increased to 4.6 mA at 10 sccm, 4.28 mA at 15 sccm, and 3.4 mA at 20 sccm, respectively. The extraction current at 1000 W and 5 sccm was 11.4 mA, decreasing to 10.2 mA at 10 sccm, 8.6 mA at 15 sccm, and 7.5 mA at 20 sccm, with each increase in the flow rate resulting in decreases of 11%, 25%, and 34%, respectively. The extraction current at 1500 W and 5 sccm was 16.5 mA, decreasing to 16.0 mA at 10 sccm, 13.5 mA at 15 sccm, and 11.6 mA at 20 sccm, with the flow rate increases leading to decreases of 3%, 18%, and 30%, respectively. At 2000 W and 5 sccm, the extraction current was 21.3 mA, decreasing by 2%, 16%, and 27% with each flow rate increase, respectively. At 2500 W and 5 sccm, the extraction current was 25.4 mA, decreasing by 3%, 15%, and 25% with each flow rate increase, respectively. At 2800 W and 5 sccm, the extraction current was 28.1 mA, decreasing by 5%, 17%, and 23% with each flow rate increase, respectively.

### 3.2. Comparison with Process Gas and IHC DC Ion Source Extraction Current Using High-Current Ion Implanter

**Figure 5:**
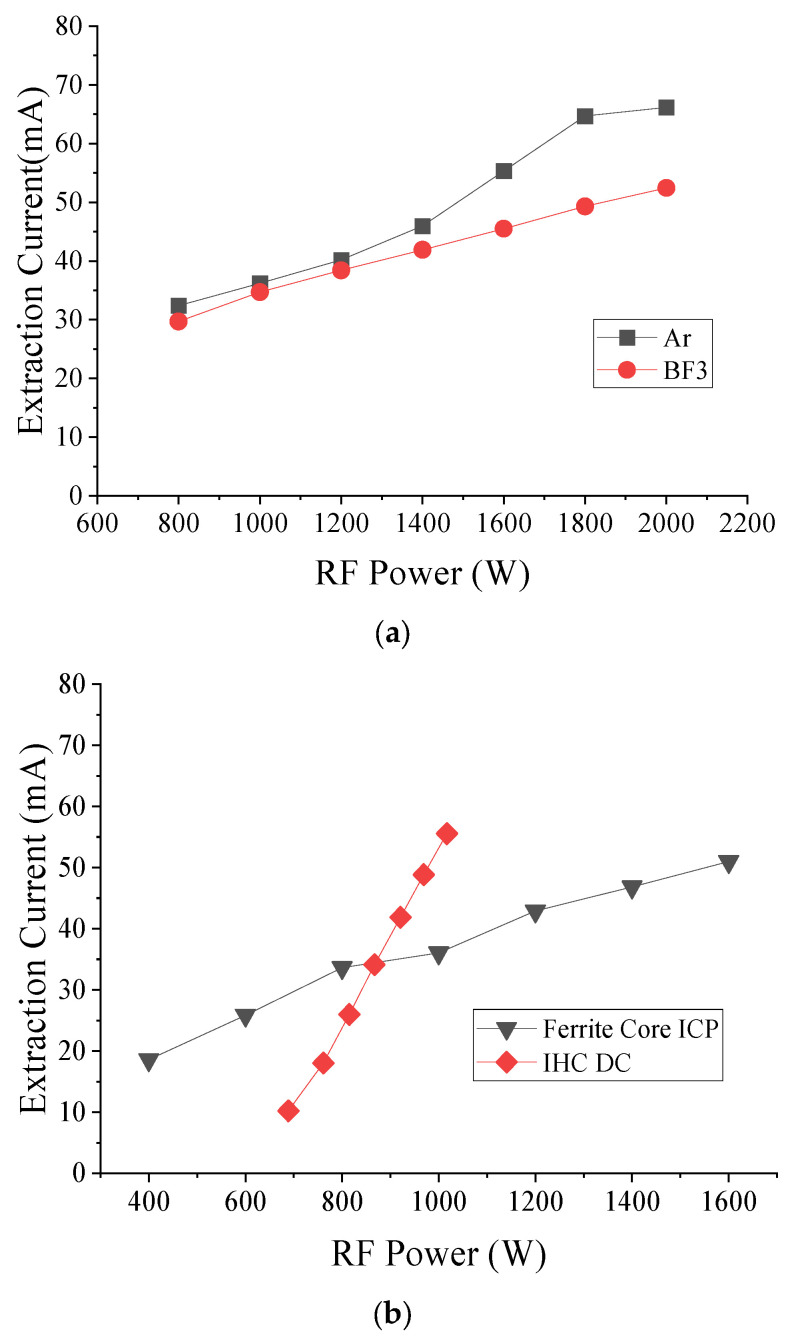
(**a**) Comparison extraction current of Ar and BF_3_ at ver. 1.2; (**b**) comparison extraction current of ver. 1.2 ferrite ICP RF and IHC DC.

The extraction current in argon gas was measured and compared with BF_3_ in a study evaluating RF power increments from 800 W to 2000 W in 200 W steps, with argon flowing at 5 sccm. The results are shown in Figure 5a. At 800 W, argon exhibited an extraction current of 32.4 mA, while BF_3_ showed 29.7 mA, representing a 9.1% higher current for argon. At 1000 W, argon reached 36.2 mA, and BF_3_ reached 34.7 mA, which is 4.1% higher. At 1200 W, argon was at 40.2 mA and BF_3_ was at 37.4 mA, which is 4.7% higher. At 1400 W, argon reached 46.0 mA and BF_3_ reached 41.9 mA, which is 9.7% higher. At 1600 W, argon was at 53.3 mA and BF_3_ at 45.5 mA, which is 21.6% higher. At 1800 W, argon reached 64.7 mA and BF_3_ reached 49.3 mA, which is 31.2% higher. At 2000 W, argon was at 66.2 mA and BF_3_ at 54.5 mA, which is 26.2% higher.

The evaluation was conducted In a commercial implanter to confirm the performance of the ferrite core ICP RF source and the IHC DC source. The evaluation conditions included an extraction voltage of 10 kV, argon flow at 5 sccm, source magnet ICP at 1.2 A, and IHC at 3.5 A. Although the source magnet was intended to be evaluated under the same conditions, ignition was not achieved at 3.5 A for the ICP, so it was decreased to 1.2 A until a stable condition was obtained.

The results, which are shown in Figure 5b, indicate that for the ICP, the extraction current was 18.57 mA at 400 W and increased to 25.9, 33.7, 36.0, 42.9, 46.8, and 51.0 mA at 1600 W, with increments of 200 W. For the IHC, the total power was calculated by summing the applied arc, cathode, and filter power supply amounts. The extraction current was 10.2 mA at 897 W, 18.0 mA at 761 W, 26.0 mA at 815 W, 34.1 mA at 867 W, 41.9 mA at 921 W, 48.8 mA at 969 W, and 55.6 mA at 1017 W. The extraction current was observed to be higher than the input power up to 880 W, but the efficiency decreased after that. The source magnet magnetic flux optimized for the shape of the IHC DC source arc chamber can explain the low ICP RF source density growth rate. Plasma was created within 100% of the source magnet flux in the IHC DC source. However, only 25% of the IHC arc chamber area was within the magnet flux in the central ferrite core.

### 3.3. Ferrite Core ICP Source Lifetime Test

For the stability test of the ICP source, we evaluated the plasma duration at an argon flow rate of 5 sccm and 2000 W. The plasma turned off during the third test after 8, 10, and 9 h, respectively. After the plasma was turned off, it could not be reignited. Upon disassembly and inspection, we found that the sub-ferrite core was broken. The inability to reignite the plasma was due to the RF power not being transmitted effectively because of the broken ferrite core. Additionally, the sub-ferrite core cable was damaged by arcing.

**Figure 6:**
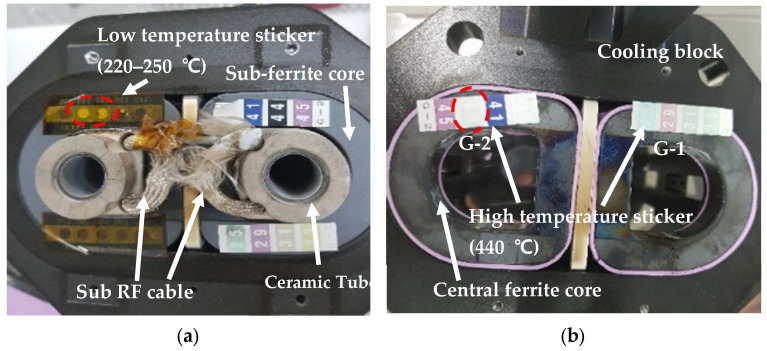
Temperature measurement of (**a**) sub-ferrite core after 30 min (When it turns from white to black, it indicates the temperature has been reached) and (**b**) central ferrite core after 8 h (When it changes from black to gray, it indicates that the temperature has reached 440 °C).

Due to these issues, we measured the temperatures at critical points of the ion source. The temperature of each point was measured using tape that changes color according to temperature (low-temperature label tape: Thermo Label 5E-210, NiGK Corporation (Saitama, Japan), high-temperature label tape: Thermo Label G-1 and G-2, NiGK Corporation). As shown in Table 1, we recorded the temperatures of the ferrite core, cable, and arc slit aperture after 30 min and 8 h of operation as shown in Figure 6. The sub-ferrite core remained below 210 °C for 30 min but exceeded 250 °C after 8 h. The central ferrite core reached 440 °C after 8 h, surpassing the 250 °C threshold, which is the maximum operating temperature for the ferrite core. The sub-ferrite core cable also exceeded 450 °C, even in the 30 min test, indicating that the cable was subjected to unsustainable temperatures of 450 °C or higher. These findings highlight the need for improved thermal management to ensure the longevity and stability of the ICP source during extended operations. 

## 4. Conclusions

This study achieved significant results using a ferrite core ICP RF ion source within an ion implanter to generate an ion beam and measure the extraction current. The sub-ferrite core enhanced the gas’s exercise energy for plasma ignition under high-vacuum conditions. As a result of measuring the ionization efficiency using a Langmuir probe, a 38% increase in ion density was observed. Subsequently, the ICP RF ion source was installed on a commercial ion implanter. High voltage was applied to form an ion beam, resulting in a 27% improvement in the extraction current.

The optimized ver. 1.1 ion source was used to evaluate the extraction current relative to the gas flow rate. It was observed that the current decreased as the gas flow rate increased, indicating that a sufficient mean free path is required for effective gas ionization. The increase in gas density in a confined space prevented sufficient energy acquisition, explaining this phenomenon.

In addition, the experimental parameters, argon ion density and extraction current, were investigated and compared with the process gas BF_3_. The ion source, equipped with additional ferrite cores (seven pairs in ver. 1.2), demonstrated a remarkably 17% higher ionization efficiency for argon than BF_3_ gas. We compared the extraction current using the same commercial ion implanter equipment to assess the equivalent performance of the IHC DC source and the ferrite core ICP RF source. Below 880 W, the ferrite core ICP source exhibited a higher extraction current, but the rate of increase diminished at higher power levels. This suggests that while the ferrite core ICP source can generate plasma at lower energies, its efficiency is less than that of the IHC DC source at higher power levels.

An on-time plasma evaluation assessed the ion source’s lifetime, but the plasma did not last beyond 10 h. Temperature measurements at critical areas revealed temperatures exceeded 450 °C, causing the ferrite core to break or the RF cable to melt. These findings indicate that the ferrite core ICP source is unsuitable for mass production. The curing temperature for producing the ferrite core is around 300 °C, and becauseferrite cores cannot withstand temperatures above 450 °C, further research utilizing ferrite cores becomes impractical. In our future studies, will focus on enhancing the plasma efficiency in the ICP RF region without relying on ferrite cores.

## Figures and Tables

**Table 1:** Temperature of ferrite core at plasma on time test.

Check Point	RF 2000 W, 8 h	RF 2000 W, 30 min
Sub-ferrite core	250 °C	210 °C
Central ferrite core	440 °C	250 °C
Arc slit aperture	450 °C	450 °C
Sub cable	450 °C	450 °C
Central cable	210 °C	210 °C

## Data Availability

The data will be made available upon request.
